# Cathelicidins: family of antimicrobial peptides. A review

**DOI:** 10.1007/s11033-012-1997-x

**Published:** 2012-10-14

**Authors:** Ewa M. Kościuczuk, Paweł Lisowski, Justyna Jarczak, Nina Strzałkowska, Artur Jóźwik, Jarosław Horbańczuk, Józef Krzyżewski, Lech Zwierzchowski, Emilia Bagnicka

**Affiliations:** Institute of Genetics and Animal Breeding in Jastrzębiec, Polish Academy of Sciences, 05-552 Magdalenka, Poland

**Keywords:** AMP, Cathelicidin, Vertebrates, Human, Farm animals, Activity, Expression

## Abstract

Cathelicidins are small, cationic, antimicrobial peptides found in humans and other species, including farm animals (cattle, horses, pigs, sheep, goats, chickens, rabbits and in some species of fish). These proteolytically activated peptides are part of the innate immune system of many vertebrates. These peptides show a broad spectrum of antimicrobial activity against bacteria, enveloped viruses and fungi. Apart from exerting direct antimicrobial effects, cathelicidins can also trigger specific defense responses in the host. Their roles in various pathophysiological conditions have been studied in mice and humans, but there are limited information about their expression sites and activities in livestock. The aim of the present review is to summarize current information about these antimicrobial peptides in farm animals, highlighting peptide expression sites, activities, and future applications for human and veterinary medicine.

## Introduction

The antimicrobial peptides are a conserved component of the innate immune response in all organisms, including plants, animals and humans. Cathelicidins, together with defensins belong to the large group of cationic peptides with amphipathic properties and represent the main part of the immune system in many vertebrates, including humans and farm animals. A lot of antimicrobial peptides (AMPs) are stored in neutrophil and macrophage granules. They are part of the oxygen-independent activity against pathogens [1]. The existence of the family of antimicrobial peptides named cathelicidins was established based on the presence of a conserved cathelin domain. The first cathelicidin, cecropin, was isolated in 1980 from tissues of the *Hyalophora cecropia* moth after a 10 year study on insect immunity [2]. Another member of the cathelicidins family, magainin, was isolated in 1987 by Zasloff from the skin of the *Xenopus leavis* frog [3]. The first mammalian cathelicidins seemed to be bactenecins isolated in the late 1980s from bovine neutrophils. These cathelicidins were named Bac5 and 7 [4].The porcine cecropin P1, isolated in the late 1980s is numbered in the cathelicidin family as well [5]. However, some authors state that the first mammalian described cathelicidin was rabbit CAP18 [6]. Till now, several cathelicidins have been identified in: cattle [4], buffalo [7] horse [8], pig [9], sheep [10], goat [11], deer [12], chicken [13] and some species of fish [14, 15], while only single peptides have been found in humans [16], rhesus monkeys [17], mice [18], rats [19], and guinea pigs [20]. Recently, a cathelicidin was also isolated from the snake *Bungarus fasciatus* [21].

The peptide family named “cathelicidins” with a common proregion (cathelin domain) were first identified in mammals in bone marrow myeloid cells [1, 22]. Therefore they are also named “myeloid antimicrobial peptides” (MAP). Cathelicidins are a group of antimicrobial peptides, varying in amino acid (a.a) sequence, structure and size. They are stored in the secretory granules of neutrophils and macrophages and can be released extracellularly upon leukocyte activation. Then, their expression was also found in non-myeolid cells for example in epithelial cells [22–24]. Cathelicidins, containing two functional domains, owe their name to the presence of the region with very high homology to cathelin—the cathepsin L inhibitor, at their N-terminal. The “cathelin” domain shows very high interspecies homology in cathelin basic aa sequence, while the antimicrobial domain located at C-terminus shows high diversity, both interspecies and intraspecies [1, 22–24].

The aim of this review is to summarize information about cathelicidins with regards to farm animals in order to provide an overview of the expression sites, activities and future application for human and veterinary medicine of these antimicrobial peptides.

## Diversity of cathelicidins

About 30 cathelicidin family members (Table 1) have been identified in mammalian species. Nevertheless, only single cathelicidins have been found in humans, rhesus monkeys, mice, rats, and guinea pigs. These cathelicidins are named LL-37, RL-37, mCRAMP, rCRAMP and CAP11, respectively [14, 16–20, 24–27]. Three cathelicidins were identified in horses (*Equus caballus* cathelicidin eCATH-1, –2 and -3) [8]. Furthermore, peptides similar to cathelicidins were described in pigs (*Sus scrofa domestica* cathelicidin PMAP37, 36 and 23, PG1, 2, 3, 4 and 5, PR39, and PF-1, -2) [28–35], cattle (*Bos taurus* cathelicidin BMAP27, 28 and 34, Bac5 and 7, indolicidin, and bactenecin-1) [36–39], buffalo (*Buballus bubalis* cathelicidin-4, myeloid cathelicidin) [7], deer (*Cervus elaphus hispanicus* cathelicidin, bactenecin) [12], sheep (*Ovis avies* cathelicidin SMAP29 and 34, OaBac5, 6, 7.5, 11) [40–42], goat (*Capra hircus* cathelicidin BAC5, BAC7.5, MAP34A and B, MAP28, ChBac3.4) [11, 43], chicken (*Gallus gallus domesticus* cathelicidin CATHL1, 2/CMAP27, 3 and Cathelicidin-B1) [13], Atlantic salmon (*Salmo salar* cathelicidin, asCath1 and 2) [44] and Rainbow trout (*Oncorhynchus mykiss* cathelicidin, rtCath1 and 2) [44]. Another fish, Atlantic hagfish (*Myxine glutinosa*) genome contains three cathelicidin-like sequences (HFIAP-1, -2, and -3) [15]. Moreover, two sheep genes: *OaDodeA* and *OaDodeB* encoded identical dodecapeptides named Cathelicidin-1 [40]

**Table 1:** Gene and protein names of cathelicidins, their function and site of expression in some species of mammals’ (*Mammalia*), birds’ (*Aves*), reptiles’ (*Reptilia*) and fish’s (*Pisces*) classes

Gene name	Peptide name	Expression site	Function	Source	Accesion number
Buffalo (*Buballus bubalis*)					
*CATH*	Cathelicidin	Female reproductive tract		[7]	Q0MX34
	Myeloid cathelicidin (fragment)	Bone marrow			Q0MX33
	Cathelicidin-4 (fragment)				C7FEV5
Banded krait (*Bungarus fasciatus*)					
	Cathelicidin-BF antimicrobial peptide	Venom gland, stomach, trachea, skin, muscle, heart, kidney, lung, brain, intestine, spleen, liver, and ovary	Potent antimicrobial peptide against some Gram-positive bacteria (*Bacillus*) and fungi	[21]	B6D434
Bovine (*Bos taurus*)					
*CATHL1* (*BAC1*)	Cathelicidin-1 (Bactenecin-1)	Large granules of neutrophils	Potent microbicidal activity against *S. aureus,* *E. coli*	[37]	P22226
*CATHL2* (*BAC5*)	Cathelicidin-2 (Bactenecin-5)	Large granules of neutrophils	Potent antimicrobial activity	[37]	P19660
*CATHL3* (*BAC7*)	Cathelicidin-3 (Bactinecin-7)	Large granules of neutrophils	Potent antimicrobial activity	[37]	P19661
*CATHL4*	Cathelicidin-4 (Indolicidin)	Cytoplasmatic granules of neutrophils	Potent microbicidal activity against *S. aureus,* *E. coli*	[38]	P33046
*CATHL5* (*BMAP28*)	Cathelicidin-5 (Antibacterial peptide BMAP-28, Myeloid antibacterial peptide 28)		Potent antimicrobial activity against Gram-negative and Gram-positive bacteria, including methicillin-resistant *S. aureus,* fungi	[37]	P54229
*CATHL6* (*BMAP27*)	Cathelicidin-6 (Antibacterial peptide BMAP27), (Myeloid antibacterial peptide27)	Bone marrow	Potent antimicrobial activity against Gram-negative and Gram-positive bacteria, including methicillin-resistant *S. aureus,* fungi	[39]	P54228
*CATHL7* (*BMAP34*)	Cathelicidin-7 (Antibacterial peptide BMAP-34)	Bone marrow cells and other tissues and organs	Potent antimicrobial activity	[36]	P56425
Goat (*Capra hircus*)					
*BAC7.5*	Bac7.5 protein	Bone marrow		[43]	Q9XSQ9
*ATHL2*	Cathelicidin-2 (Bactenecin-5)	Bone marrow and leukocytes	Binds to the LPS of all Gram-negative bacteria. Potent antimicrobial activity against Gram-negative bacteria: *S.typhimurium,* *P. aeruginosa, E. coli*. Less active against Gram-positive bacteria: *S.aureus*, *L. monocytogenes, B. subtilis*	[11]	P82018
*MAP28*	MAP28 protein	Bone marrow			Q9XSQ8
*MAP34*-*A*, *MAP34*-*B*	MAP34-A protein or MAP34-B protein	Bone marrow			P82017
	Cathelicidin-3.4 (Bactenecin-3.4)	Leukocytes	Potent antibacterial activity against Gram-negative bacteria *E. coli,* *P. aeruginosa*. Less active against Gram-positive bacteria: *S.aureus,* *L. monocytogenes*. Low hemolytic activity towards human erythrocytes		P85170
Guinea pig (*Cavia porcellus*)					
*CAP11*	Neutrophil cationic antibacterial polypeptide of 11 kDa	Granules of neutrophils		[20]	Q91X12
Red Deer (*Cervus elaphus hispanicus*)					
	Bactenecin	Neutrophils	Strong activity against Gram-negative bacteria, lower activity against Gram-positive bacteria and yeast	[12, 36]	A8QJ91
Horse (*Equus caballus*)					
*eCATH*-*1*	Myeloid cathelicidin 1	Bone marrow		[8]	O62840
*eCATH*-*2*	Myeloid cathelicidin 2	Bone marrow		[8]	O62841
*eCATH*-*3*	Myeloid cathelicidin 3	Bone marrow		[8]	O62842
Chicken (*Gallus gallus domesticus*)					
*CATHL1*	Cathelicidin-1	Gizzard, liver, small intestine, large intestine, cloaca, bursa of Fabricius, gall bladder, lung, trachea, kidney, testis and bone marrow	Binds to the LPS. Potent antimicrobial activity against Gram-positive and Gram-negative bacteria, hemolytic activity (in vitro)	[13]	Q6QLQ5
*CATHL2* (*CMAP27*)	Cathelicidin-2	Trachea, lung, proventriculus, duodenum, jejunum, ileum, caeca, colon, caecal tonsil, bursa of Fabricius, kidney, ovary, testis, thymus, liver, spleen, bone marrow, skin, uropygial gland, muscle and brain	Binds to the LPS. Potent antimicrobial activity against Gram-positive and Gram-negative bacteria, hemolytic activity (in vitro)	[48]	Q2IAL7
*CATHL3*	Cathelicidin-3	Bone marrow, liver and lung	Binds to the LPS. Potent antimicrobial activity	[13]	Q2IAL6
*CATHB1*	Cathelicidin-B1	Bursa of Fabricius	Potent antimicrobial activity against Gram-positive and Gram-negative bacteria (in vitro)	[49]	Q5F378
Human (*Homo sapiens*)					
*CAMP* (*CAP18*, *FALL39*)	Cathelicidin antimicrobial peptide (CAP-18, hCAP-18)	Bone marrow, testes and neutrophils	Binds to LPS, antibacterial activity	[16]	P49913
Rhesus macaque (*Macaca mulatta*)					
*CAMP* (*CAP18, FALL39*)	Cathelicidin antimicrobial peptide (CAP-18, rhCAP-18)	Epithelia of various organs such as organs of the respiratory or gastrointestinal tract	Binds to LPS, antibacterial activity	[17]	Q9GLV5
Mouse (*Mus musculus*)					
*Camp* (*Cramp*)	Cathelin-related antimicrobial peptide (Cramp)	Testis, spleen, stomach, and intestine	Acts as a potent antimicrobial peptide	[18]	P51437
Atlantic hagfish (*Myxine glutinosa*)					
*CATH29*	Hematopoietic antimicrobial peptide-29 (similar to HFIAP3)	Intestinal tissues	Potent activity against Gram-negative and Gram-positive bacteria	[15]	Q71MD5
*CATH37*	Hematopoietic antimicrobial peptide-37(similar to HFIAP 1,2)	Intestinal tissues	Potent activity against Gram-negative and Gram-positive bacteria	[15]	Q71MD7
Rabbit (*Oryctolagus cuniculus*)					
*CAP18*	Antimicrobial protein CAP18	Neutrophils	Binds to the LPS of all Gram-negative bacteria, antibiotic activity	[50]	P25230
*P15R*	Protein P15A	Large granules of neutrophils.	Binds to the LPS, potentiates strongly the early antibacterial effects of bactericidal/permeability-increasing protein (BPI)	[51]	P26202
*P15H*	Protein P15B	Large granules of neutrophils.	Binds to the LPS, potentiates weakly the early antibacterial effects of BPI	[51]	P26203
Sheep (*Ovis avies*)					
*BAC6*	Bactinecin-6			[40]	O19040
*BAC7.5*	Bactinecin-7.5			[41]	P79361
*BAC11*	Bactinecin-11			[40]	O19031
*CATHL1A* (*OaDodeA*) and *CATHL1B* (*OaDodeB*)	Cathelicidin-1(Bactenecin-1)		Potent microbicidal activity; active against *S. aureus* and *E. coli*	[40]	P54230
*CATHL2* (*BAC5*)	Cathelicidin-2(Bactenecin-5)	Liver	Binds to the LPS of all Gram-negative bacteria, potent antimicrobial activity	[40]	P79362
*CATHL3* (*BAC7*)	Cathelicidin-3 (Bactenecin-7)	Bone marrow	Potent antimicrobial activity	[42]	P50415
*SMAP*-*29* (*SC5*)	Cathelin-related peptide SC5 (Myeloid antibacterial peptide MAP-29)	Liver, bone marrow	Broad spectrum bactericidal agent	[41]	P49928
*MAP34*	Myeloid antimicrobial peptide	Liver		[41]	P79360
Rainbow trout (*Oncorhynchus* *mykiss*)					
*rtCATH*-*1*	Cathelicidin-derived antimicrobial peptide-1	Gill, head kidney and spleen		[44]	Q49S73
*rtCATH*-*2*	Cathelicidin-derived antimicrobial peptide-2	Gill, head kidney, intestine, skin and spleen		[44]	Q2KSZ4
Rat (*Rattus norvegicus*)					
*Camp*	Cathelicidin antimicrobial peptide (rCRAMP)			[19]	Q71KM5
Atlantic salmon (*Salmo salar*)					
*asCATH*	Cathepsin H			[44]	B5X7S5
*asCATH*-*2*	Cathelicidin 2			[44]	Q49TU5
Pig (*Sus scrofa domestica*)					
*PMAP23*	Antibacterial peptide PMAP-23 (Myeloid antibacterial peptide 23)	Bone marrow and liver	Antimicrobial activity against Gram-positive and Gram-negative bacteria	[28]	P49930
*PMAP36*	Antibacterial peptide PMAP-36 (Myeloid antibacterial peptide 36)	Bone marrow	Antimicrobial activity against Gram-positive and Gram-negative bacteria	[29]	P49931
*PMAP37*	Antibacterial peptide PMAP-37 (Myeloid antibacterial peptide 37)	Bone marrow	Antimicrobial activity against Gram-positive and Gram-negative bacteria	[30]	P49932
*PR39*	Antibacterial protein PR-39	Small intestine and bone marrow	Potent antimicrobial activity against *E. coli* and *B. megaterium*	[31]	P80054
	Cathelin	Leukocytes	Probably a microbicidal peptide	[32]	P15175
*PF*-*1*	Prophenin-1	Bone marrow and leukocytes	Antimicrobial activity against Gram-negative bacteria (more effective) and Gram-positive bacteria (less active)	[33]	P51524
*PF*-*2*	Prophenin-2	Bone marrow and liver	Antimicrobial activity against Gram-negative bacteria (more effective) and Gram-positive bacteria (less active)	[34]	P51525
*NPG1*	Protegrin-1	Bone marrow, leukocytes and neutrophils	Microbicidal activity against *L. monocytogenes, E. coli* and *C. albicans*, (in vitro)	[35]	P32194
*NPG2*	Protegrin-2	Bone marrow, leukocytes and neutrophils	Microbicidal activity against *L. monocytogenes, E. coli* and *C. albicans*, in vitro	[9]	P32195
*NPG3*	Protegrin-3	Bone marrow, leukocytes and neutrophils	Microbicidal activity against *L. monocytogenes, E. coli* and *C. albicans*, in vitro	[52]	P32196
*NPG4*	Protegrin-4	Bone marrow, leukocytes and neutrophils	Microbicidal activity	[53]	P49933
*NPG5*	Protegrin-5	Bone marrow, leukocytes and neutrophils	Microbicidal activity	[54]	P49934

Accession number derives from UniProtKB

**Fig. 1:**
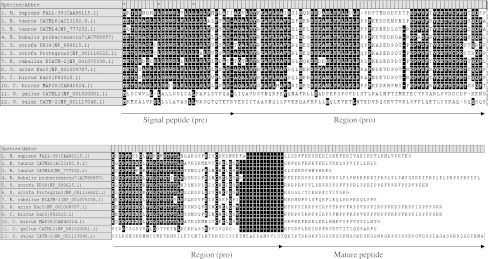
Alignment of amino acid sequences of vertebrate’s cathelicidins. In the left column is shown: species name, name of cathelicidin with accession no. (GenBank). Conserved site with at least 60 % level of sequence identity are marked

Cathelicidins are stored in neutrophil granules as inactive precursors (prepropeptides). They are released as mature peptides when required, after being cleaved by neutrophil elastase [45]. The N-terminal signal sequence and proregion (cathelin) are highly conserved among species and different peptides, whereas the C-terminal domain, encoding the mature peptide, shows substantial heterogeneity (Fig. 1) [22, 24]. The C-terminal domains in some cathelicidin peptides are α-helical, in others β-hairpin, and in some peptides they are proline/arginine rich. The mature peptide ranges in size from 12 to 80 or more aa residues [22]. The cathelicidin family consists of five distinct groups of peptides [24]. They comprise: (1) cyclic dodecapeptides with one disulfide bond, (2) porcine protegrins with two disulfide bonds, (3) peptides with α-helical structure such as bovine BMAP-27, -28 and -34, ovine SMAP-29 and -34, porcine PMAP-23, -36 and -37, human hCAP18 and the three known equine cathelicidins, (4) peptides containing a high number of tryptophan residues such as indolicidin and peptides containing a high number of proline and arginine residues, and (5) short molecules arranged in tandem repeats such as bactenecins (bovine Bac5 and 7, ovine OaBac5 and 7.5 as well as porcine PR-39 and prophenins) [24, 25, 46]. In the bovine bactenecin, three tandem repeats of a tetradecamer composed of several Pro–Arg–Pro triplets were found. Between triplets, a single hydrophobic aa residue occurs. Three repeats of the decamer FPPPNFPGPR were also found in pig prophenins [47].

All cathelicidins are encoded by genes consisting of four exons. The first exon covers a sequence encoding the signal peptide (part pre-) of 29–30 aa residues in size, while exons 2 and 3 encode the cathelin domain (part pro-) of 99–114 aa. Exon 4 encodes the mature peptide, with the antimicrobial domain consisting of 12–100 aa. Till now, on sheep, cow, pig and chicken chromosomes, all cathelicidin genes were found to form clusters [13, 55, 56]. Cathelicidin genes were localized on chromosome 13 in pig, chromosome 19 in sheep (8 genes), and in chickens on chromosome 2p at the proximal end as a dense cluster within a 7.5-kb (3 genes) [13, 31, 40]. In cattle, more than 10 cathelicidin genes were located in the same region on chromosome 22q24 [36]. In human and mouse genome cathelicidin genes were localized on chromosomes 3 and 9, respectively [31].

Phylogenetic analysis demonstrated that genes encoding chicken cathelicidins and mammalian neutrophilic granule peptides are probably descendants from a single, remotely related gene, evolved prior to the separation of birds from mammals. However, it is supposed that genes encoding other “classic” mammalian cathelicidins (cathelicidins with high homology in cathelin domain) may have been duplicated from the ancestral neutrophilic granule peptide gene after mammals and birds drifted apart [13].

## Human cathelicidin

Despite the existence of a great amount of beta defensin (hBD) genes in human genome reviewed in [57, 58], there is only one cathelicidin gene (*CAMP*) identified in humans. *CAMP* encodes the peptide LL-37 which begins with two leucine residues at its N-terminus, and is 37 aa residues long, with a molecular weight of 18 kDa [31]. It is also known as hCAP-18, FALL-39 or CAMP—human cationic antimicrobial peptide. LL-37 is expressed in various cells and tissues such as circulating neutrophils and myeloid bone marrow cells, epithelial cells of the skin, and is also expressed in the gastrointestinal tract, as well as in the epididymis and lungs. Expression was also detected in squamous epithelium of the mouth, tongue, esophagus and in the colonic and bronchial mucosal epithelium [59, 60]. Moreover, production of LL-37 in macrophages is stimulated by vitamin D released by sunlight through the skin. Probably the sun baths, recommended for years for overcoming tuberculosis, increase the ability of LL-37 to kill intracellular *Mycobacterium tuberculosis* [61]. Transcripts of *CAMP* gene were isolated from the lung, genitourinary tract, skin keratinocytes in inflammatory disorders, as well as from B cells, T cells, natural killer cells, monocytes, and macrophages [62]. Expression of LL-37 could be constitutive or inducible by microbial, inflammatory, and developmental stimulation. Nevertheless, the knowledge of molecular mechanisms of gene regulation is still very limited [24]. A few potential binding sites for transcription factors which probably regulate *CAMP* gene expression were found in the human cathelicidin gene [63]. Human cathelicidin acts in the promotion of wound healing, as direct and indirect antimicrobial factors and can also modulate the adaptive immunity [64].

## Bovine cathelicidins

The first bovine cathelicidins isolated from neutrophils were bactenecins 5 and 7; abbreviated as Bac5 and Bac7. The term bactenecin was created from two words: “*bacterium*” and the Latin word “*necare*” that means to kill. These peptides consist of 43 and 60 aa residues, respectively, with a different, polycationic aa sequence, but both characterized by a repeated proline motif [4]. These proline-rich antimicrobial peptides have been reported to kill bacteria without significant membrane lysis. These peptides generally show higher selectivity for Gram-negative than for Gram-positive bacteria [65]. The bactenecin expressed by bovine neutrophils (Bac5) has a β-hairpin structure with four arginine residues and one intramolecular disulfide bond. The homodimeric form of bactenecin shows higher antibacterial activity with less sensitivity to salt concentration than the monomer. Both the mono- and dimeric forms of bactenecins kill *Staphyloccocus aureus* at concentrations of 8–16 μM within 10–30 min. The differences in the structure of peptide forms cause differences in their mode of action. The homodimeric bactenecins form pores in the pathogen’s membrane to disrupt the cells, whereas the targets of monomeric peptide are intracellular organelles. As a result, this peptide inhibits some functions such as synthesis of cell wall, proteins or nucleic acids [66]. The cDNA encoding Bac5 clone was used to prove that dodecapeptide bactenecins and other structurally unrelated antimicrobial peptides of the bovine, despite their diversity, are closely related since their transcripts showed a high degree of nucleotide sequence similarity [22, 67].

The bovine BMAP-27 and BMAP-28 (bovine myeloid antimicrobial peptides of 27 and 28 aa residues, respectively) contain α-helical C-terminus with structural attributes of antimicrobial activity. Owing to its direct antimicrobial activity and modulating the inflammatory response, BMAP-28 probably supports the host defense. At low concentrations in vitro, bovine BMAP-28 not only kills bacteria and fungi, but is also toxic for mammalian tumor cells, inducing their apoptosis [39, 68]. Moreover, this AMP shows cytotoxic activity against other mammalian cells. It was also demonstrated that BMAP 28 induces mitochondrial permeability forming transition pores (MPTP) resulting in the release of *cytochrome* c [69]. These peptides showed high effectiveness against pathogens in mastitic bovine milk and low efficiency in milk from healthy cows. BMAPs may also activate the immune response by stimulating the expression of tumor necrosis factor alpha (TNF-α) in bovine mammary epithelial cells [65].

Another bovine cathelicidin is indolicidin. It is a tryptophan-rich peptide of 13 aa (ILPWKWPWWPWRR-NH2), purified from the cytoplasmic granules of neutrophils [38]. Moreover, Del Sal et al. [70] reported that this peptide is synthesized in bone marrow cells as 144 aa-long precursor. Indolicidin shows activity against different species of pathogenic fungi like *Candida albicans,* *Cryptococcus neoformans,* bacteria—*S. aureus,* and *E. coli* [38, 71]. This AMP exhibits a destructive effect on intracellular targets, such as bacterial DNA and RNA [65, 72] and it is capable of inducing an autophagic cell death in the protozoan pathogen *Leishmania donovani* [73], as well as killing the trophozoites—*Giardia lam* [74].

## Porcine cathelicidins

Until now, five small (16–18 aa residues) cathelicidins named protegrins, with a β-hairpin structure stabilized by two intramolecular disulfide bonds between cysteines, were found in porcine bone marrow and neutrophils. Protegrins display limited sequence similarity to certain defensins and tachyplesins [75]. They have antimicrobial activity against bacteria, especially Gram-negative, fungi, and some enveloped viruses when used at concentrations of 1–5 μg/ml [9, 76, 77]. Protegrin-1 (PG-1; 18 aa), consisting of 6 arginine residues, has antiparallel β-sheet structure, protegrin-2 is two aa residues shorter and has one less positive charge than in PG-1. Protegrin 3 has glycine instead of arginine at position 4 which also causes one less positive charge than PG-1. PG-4 has phenylalanine instead of valine at position 14 and this substitution caused the difference in the β-turn. This difference makes PG-4 less polar and less positively charged than other peptides. The fifth porcine protegrin—PG-5 has a substitution proline–arginine at position 10 and one less positive charge than PG-1 [78].

The gene structures of three other porcine cathelicidins have been characterized by Gudmundsson et al. and Zhao et al. [31, 34, 54]. The signal sequence of 29 residues and the first 37 residues of the cathelin propart are contained in the first exon. Exons 2 and 3 contain cathelin information, and the C-terminal domain, encoding the mature peptide PR-39 extended by three residues. PR-39 has multiple activities contributing to the innate defense of pigs. It is a proline–arginine-rich antibacterial peptide that was isolated originally from the porcine small intestine, and subsequently localized in neutrophils. PR-39 enters cells without membrane lysis, and after a short lag is capable of killing bacteria by inhibiting bacterial messenger RNA translation and DNA synthesis [79]. It was also been reported that PR-39 modulates production of proteoglycans in wound healing, to promote leukocyte chemotaxis, to interact with sarcoma homology 3 domain (SH3) of different proteins, and to inhibit superoxide production by neutrophils [79]. Increased expressions of PR-39 and protegrins in porcine bone marrow progenitor cells have been observed following cell activation with bacteria or purified lipopolysaccharides (LPS) [80].

Prophenin-1 (PF-1), the peptide with a molecular mass of 8,683 Da and 79 aa residues (42 prolines and 15 phenylalanines), was isolated from pig leukocytes. Three perfect and three nearly perfect repeats of a decamer, FPPPNFPGPR were found in Prophenin-1 N-terminal. Prophenin-2 (PF-2), with 97 aa residues, stored in secondary granules of neutrophils, is expressed in immature myeloid cells. Both peptides in vitro show more activity against *E. coli*, than against *Listeria monocytogenes* (Gram-positive bacteria) [47, 81].

## Caprine and ovine cathelicidins

There are not many studies describing cathelicidins in sheep and goats. Until now, at least eight cathelin-associated peptides, were identified in sheep, including cyclic dodecapeptide, SMAP29 and Bac5, 6, and 7.5, but little is known about their antimicrobial properties [11, 43]. Four of eight cathelicidin genes encode the proline- and arginine-rich peptides named OaBac5, 6, 7.5 and 11 [40], while SMAP29 is an α-helical peptide [43].

In goats, a peptide named ChBac5, with almost exclusively X–P–P–Y repeats, was identified and Bac7.5 as well. Shamova et al. [11] showed that Bac5 peptides from sheep and goats bind to bacterial lipopolysaccharides (LPS), a glycolipid present in the outer membrane of all Gram-negative bacteria, and Bac5 kills them at concentrations of NaCl similar to those found in extracellular fluids. Both cathelicidins show high activity against all microbes studied at a low salt concentration, whereas at a high salt concentration (100 mM NaCl) these peptides are still active against Gram-negative bacteria (*E. coli*, *Bacillus subtilis* and *Pseudomonas aeruginosa*), but have no activity against *S. aureus*, and *C. albicans.* Anderson et al. [43] found that SMAP29 and other ovine cathelicidins: OaBac5mini and OaBac7.5mini showed activity against Gram-negative, Gram-positive bacteria as well as *C. albicans* at minimum inhibitory concentration (MIC) between 0.125 and 64 μg/ml, depending on the species of the microorganism. The study of Shamova et al. [82] identified in goat leukocytes, the proline-rich bactenecin—ChBac3.4 (approximate mass 3.4 kDa), which had over 50 % identity to caprine, ovine and bovine Bac5 peptides. This cathelicidin has a high ability to damage microbial membranes (*E. coli, P. aeruginosa* and *L. monocytogenes* but with reduced efficacy against *S. aureus, C. albicans*), especially at a low salt concentration (10 mM phosphate buffer).

## Chicken cathelicidins

Initially, there were three cathelicidins found in chickens (fowlicidin-1, -2 and -3, also known as chCATH-1, -2 and -3). Then, chCATH-B1 with an antibacterial activity has been discovered [83]. Fowlicidins show activity against Gram-negative and Gram-positive bacteria, including antibiotic-resistant strains. The activity of fowlicidins’ MIC varied between 0.4 and 2.0 μM for most strains, and activity did not depend on salt concentration [13]. The expression of these peptides was identified first in the bursa Fabricius. [84] The chicken cathelicidin B1 (chCATH-B1) in particular, is expressed exclusively in bursal epithelial cells. The C-terminus (cathelin region) of chCATH-B1 has less homology to the mammalian cathelicidins than that of chCATH-1, -2, and -3, [13, 48]. Van Dijk et al. [48] identified in chicken bone marrow cells the peptide CMAP27 (chicken myeloid antimicrobial peptide 27), that may play a role in chicken innate defense. It appeared to be similar to bovine myeloid antimicrobial peptides (BMAP-27, BMAP-28, BMAP-34).

## Horse cathelicidins

Till now, only three cathelicidins have been identified, and those found (discovered) by Scocchi and co-workers [8] are stored in equine neutrophils. The mature peptides were found in inflammation sites, thus the processing of these propeptides probably takes place during neutrophil activation. A broad spectrum of antimicrobial activity was demonstrated for eCATH-1 and eCATH-3, while the equine cathelicidin, eCATH-2 had antibacterial activity restricted to *E. coli*, *S. aureus*. Furthermore, eCATH-3 showed potent activity against some fungi like *C. neoformans* and *Rhodotorula rubra*, but its activity depends on the salt concentration being strongly inhibited at the physiological salt concentration [8, 85].

## Fish cathelicidins

Several antimicrobial peptides, similar to mammalian cathelicidins, have been identified in fish [15, 44]. The cathelicidin found in Rainbow trout (*Oncorhynchus mykiss*) was the first identified in vertebrates outside the mammalian species [25]. The properties of fish cathelicidins have not been yet studied and there is limited information as to whether they function as a part of the immune system of fish. Atlantic cod (*Gadus morhua*) was found to have at least three cathelicidin genes. Two of them show difference in the 5′-region (N-terminal peptide region). The mature peptides of Atlantic cod contain mainly arginine, glycine and serine residues, therefore they form a novel class of peptides [86]. Cathelicidin genes in Arctic char (*Salvelinus alpinus*) and Brook trout (*Salvelinus fontinalis*) have an exon deletion in the cathelin coding region, which may result in the deletion of the predicted loop 2 of the cathelin region and its adjacent beta-strands [86]. Infection of Arctic char and Atlantic cod with pathogenic bacteria led to an increased expression of the cathelicidins hence, these peptides may play an important role in fish immunity [87].

Both Rainbow trout (*Oncorhynchus* *mykiss*) and Atlantic salmon (*Salmo salar*) have two cathelicidin genes each (named rtCath 1 and 2 and asCath 1 and 2, respectively). The inducible expression of cathelicidin 1 (rtCath1) was shown only after infection, while the constitutive expression of rtCath2 in many tissues was found in rainbow trout. The expression of rtCath2, however, was further upregulated after bacterial infection. The in vitro studies strongly suggest antibacterial activities of rtCath 1 and 2 [44]. The mature rainbow trout cathelicidins are 66-a.a long and contain 6 proline residues which form PGGGS repeats. The mature peptides of cathelicidins found in hagfish (HFIAP-1, 2 and 3) are alpha-helical peptides with a potent activity against Gram-negative and Gram-positive bacteria. Their conserved cathelin region contains four cysteine residues like in trout, chickens and many mammalian cathelicidins [25].

The presence of cathelicidin family members in some species of fish could mean that cathelicidin ancestors subsisted over 300 million years ago [56].

## Mechanisms of action against pathogens and other functions

The majority of antimicrobial peptides belonging to a large class of cationic peptides, are amphophilic. This property is needed to permeate the membranes of a pathogen. The electrostatic interaction between the cationic peptide and negatively charged membrane of bacteria is probably due to the presence of its amphophilic/amphipatic properties. The hydrophilic region causes the correct alignment of the peptide on a pathogen membrane [88, 89].

The general rule of the mechanism triggering cathelicidin action, like that of other antimicrobial peptides, involves the disintegration (damaging and puncturing) of cell membranes of organisms toward which the peptide is active. Cathelicidins do not act on healthy host cell membrane. Interaction of cationic peptides and negatively charged lipid membranes of microorganisms enable their accurate, parallel adhesion and anchoring, and neutralizing the membrane charge [77]. Changing of the secondary and tertiary structure of the peptide changes its perpendicular orientation, thus embedding in the lipid bilayer and creating transmembrane pores. In its action against Gram–negative bacteria, the peptide can move across the outer membrane, and after passing the layer of peptidoglycan, crosses the inner membrane into the cytoplasm of the bacterial cell [90, 91]. Currently, several mechanisms of peptide penetration across the cytoplasmatic membrane are known. One of them is called the “barrel stave” mechanism, based on the growing of peptides in the form of barrel staves, of which the hydrophilic inner surface creates a gap [92]. Another mechanism was named the “connecting channels”, when peptides combine with the cytoplasmic membrane and create clusters which penetrate into the interior of the cell by creating gaps [93]. It should be noted that cathelicidins, beyond the mechanisms of membrane binding, can also activate the extracellular factors that induce autolysing phospholipase A2 [94]. Porcine cathelicidins PR-39, indolicidin and synthetic peptide PR-26 were shown to inhibit protein synthesis and to induce the degradation of certain proteins needed for DNA replication of the pathogen [92]. The fungicidal activity of one bovine cathelicidin, indolicidin, involves disruption of cell membranes via direct interaction with the lipid bilayers in a salt-dependent and energy-independent manner [95]. Indolicidin can bind DNA with the sequence-preference, which may contribute to indolicidin antimicrobial action and can also inhibit topoisomerase 1, which cuts one strand of double-stranded DNA, relaxing and re-annealing the strands [96, 97].

Some actions of cathelicidins are mediated though their interaction with other cells which are the important part of innate immune system, like monocytes, dendritic cells, T cells and epithelial cells [98]. The susceptibility to bacterial infections of animals and humans with lowered expression of antimicrobial peptides shows their crucial role in the immune response. For this reason, there is a growing body of evidence suggesting that the immunomodulatory properties of the antimicrobial peptides—defensins and cathelicidins—might be used for the development of novel therapeutic agents [57, 98].

The fungicidal activity of some bovine and porcine cathelicidins was proved in the in vitro study. SMAP-29, BMAP-27 and -28, bovine indolicidin, as well as porcine PG-1 showed activity against many clinical isolates of fungi including those resistant to conventional medicines used in human therapy. All of those peptide caused the disruption of the fungi cell membrane [71]. Both human LL-37 and mouse mCRAMP cathelicidins showed similar pH-dependent activity against *C. albicans* at MIC between 15 and 20 μM (the lower pH the higher growth inhibition of *C. albicans*). However, porcine PR-39 was shown to be inactive against fungi [99].

Some information about immunomodulatory activities of the cathelicidin—bovine indolicidin, was reported by Bowdish et al. [64]. According to their studies, indolicidin inhibits secretion of TNF-alpha from macrophages in response to LPS treatment and induces the production of the chemokine interleukin-8 (IL8) in the human bronchial cell line. The other known biological function of cathelicidins is their influence on wound repair and angiogenesis, chemotaxis and antisepsis activity as well as induction of cytolysis, especially of the hematopoietic cell line and proliferating lymphocytes. Owing to inhibition of NADPH oxidase activity that generates reactive oxygen species, PR-39 prevents tissue injury [77].

## Perspectives

Intensive work on antimicrobial peptides is carried out all over the world in different scientific institutions as well as in pharmaceutical and biotechnological companies. Gene therapy through augmenting the level of cathelicidins was investigated but there has been little progress in this work [100, 101]. Currently, the possibility of applying the aerosolized protegrin directly into the lungs of patients with cystic fibrosis is being evaluated [102]. Up to 100 % systemic protection against infections caused by intraperitoneal injection of *P. aeruginosa*, *S. aureus* and methicillin-resistant *S. aureus* was conferred in clinical tests in rats by PG-1 (pig peptide protegrin) [90]. Ovine cathelicidins SMAP29 and SMAP34 are probably candidates for use in human therapy against bacterial infection and immunocompromised persons. For example, SMAP 29 is highly effective against infections causing low hydratation of respiratory airway surface liquid during cystic fibrosis lung disease. The peptide was effective under both low and high NaCl concentration. Therefore, it can be used to design of an artificial salt tolerant peptide antibiotics [103]. The salt-resistant, antimicrobial properties of CAP18 and SMAP29 suggest potential for the treatment of bacterial infections in individuals with cystic fibrosis, who have high levels of sodium chloride in the sweat [103, 104]. The in vitro study on mouse bone marrow-derived dendritic cells as well as the in vivo study on cathelicidin-deficient mice, showed that local administration of synthetic human and murine cathelicidins inhibited the allergic response. All the above listed studies confirm the ability of synthetic cathelicidins to kill or to inhibit microbes. Thus, the therapeutic use of cathelicidins or modulators of their expression is to be considered [105].

Many reports about production and activity of synthetic antimicrobial peptides appear only in the patent literature. Researchers try to obtain peptides that are shorter than natural ones but with the same or even higher activity against microbes. [106, 107] For example, 12- to 14-aa peptides like bactenecin and indolicidin derivatives were shown to have excellent broad-spectrum antimicrobial activities [106]. Also the synthetic peptide Rev4, designed based on indolicidin, showed high antimicrobial activity and improved protease resistance, since this peptide also appeared a potent inhibitor of different types of proteases [108].

Different strategies for using AMPs are taken into account. One of them is the use of a single anti-infective agent. Another possibility is taking advantage of the synergistic or additive effects of antimicrobial peptides and conventional antibiotics, or exploiting their immunostimulatory effects. The use of AMPs as endotoxin-neutralizing agents is also predicted [109]. The main advantages of using antimicrobial peptides are their broad spectrum of activity and fast action. Probably, the AMPs will show a low level of induced resistance in pathogens. However, the high costs of their synthesis, screening and manufacturing, and also the natural resistance of pathogens are the main disadvantages. Moreover, the susceptibility to proteolysis, reduced activity in physiological salts, serum, and pH sensitivity, and confounding biological functions (e.g. angiogenesis) should be taken into account. Gene therapy aimed at increasing the expression of the antimicrobial peptides in a patient’s tissues is other possible strategy which may be used. However, the fault of many viruses used as vectors in gene therapies is the potential possibility to cause inflammation. Increasing the expression of antimicrobial peptides by supplementing the diet with presumed regulators of their secretion seems to be the better option. The results of preliminary study on the influence of goat diet supplementation by yeast on expression of AMPs genes in milk somatic cells seem to indicate a positive influence of such supplementation on expression of one cathelicidin gene (Jarczak, personal communication). Nowadays, several peptides seem promising for possible drug development in preclinical studies [57, 109–112]. Despite the large amount of additional information about the cathelicidins in mammals, there are still limited information on their multiple functions. It is important to recognize novel epigenetic mechanisms that control the tissue-specific expression of the AMP genes, in order to develop novel therapeutic strategies intended to potentiate endogenous production of these molecules. To fully understand the functional potential of cathelicidins in livestock, we need to precisely understand their in vivo role. This role, however, is still unclear in domestic species.

Further studies are needed to determine the transcriptomic patterns of particular cathelicidins in particular time points of infection to unravel their role in disease. Furthermore, there is a need to develop mouse models where genes encoding AMPs are either knocked-down or overactive to see if AMPs can influence an animal’s physiology and determine how these effects impact host physiology and pathology. Screening for epigenetic effects on gene expression that can be altered in such models is also required. Other animal models (goat, bovine) are also required.
